# Genetic polymorphisms of VIP variants in the Tajik ethnic group of northwest China

**DOI:** 10.1186/s12863-014-0102-y

**Published:** 2014-09-30

**Authors:** Jiayi Zhang, Tianbo Jin, Zulfiya Yunus, Xiaolan Li, Tingting Geng, Hong Wang, Yali Cui, Chao Chen

**Affiliations:** School of Life Sciences, Northwest University, Mailbox 386, #229 North Taibai Road, Xi’an, 710069 Shaanxi China; National Engineering Research Center for Miniaturized Detection Systems, Xi’an, 710069 China; College of Life Sciences and Technology, Xinjiang University, Urumqi, 830046 China

**Keywords:** Pharmacogenomics, Genetic polymorphism, Haplotype, Tajik, Ethnic difference

## Abstract

**Background:**

Individual response to medications varies significantly among different populations, and great progress in understanding the molecular basis of drug action has been made in the past 50 years. The field of pharmacogenomics seeks to elucidate inherited differences in drug disposition and effects. While we know that different populations and ethnic groups are genetically heterogeneous, we have not found any pharmacogenomics information regarding minority groups, such as the Tajik ethnic group in northwest China.

**Results:**

We genotyped 85 Very Important Pharmacogene (VIP) variants selected from PharmGKB in 100 unrelated, healthy Tajiks from the Xinjiang Uygur Autonomous Region and compared our data with HapMap data from four major populations around the world: Han Chinese (CHB), Japanese in Tokyo (JPT), Utah Residents with Northern and Western European Ancestry (CEU), and Yorubia in Ibadan, Nigeria (YRI). We found that Tajiks differed from CHB, JPT and YRI in 30, 32, and 32 of the selected VIP genotypes respectively (*p* < 0.005), while differences between Tajiks and CEU were found in only 6 of the genotypes (*p* < 0.005). Haplotype analysis also demonstrated differences between the Tajiks and the other four populations.

**Conclusion:**

Our results contribute to the pharmacogenomics database of the Tajik ethnic group and provide a theoretical basis for safer drug administration that may be useful for diagnosing and treating disease in this population.

**Electronic supplementary material:**

The online version of this article (doi:10.1186/s12863-014-0102-y) contains supplementary material, which is available to authorized users.

## Background

To date, pharmacogenomic studies have focused on candidate genes involved in drug pharmacokinetics or pharmacodynamics. Many of these genes contain functional polymorphisms that are obvious pharmacological choices for investigation in appropriate clinical populations [1,2]. For some drugs, genetic information is important to avoid drug toxicity and to optimize response [2,3]. Pharmacogenomic studies are rapidly elucidating the inherited nature of differences in drug disposition and effects, thereby enhancing drug discovery and providing a stronger scientific basis for optimizing drug therapy on an individual basis [4].

Tajiks are an ethnic group with a worldwide population of 15 to 20 million; they live mostly in Tajikistan, Afghanistan, Uzbekistan, and the Xinjiang Uygur Autonomous Region [4]. According to the 2010 census, approximately 51,000 Tajiks live in China, mostly in the Tashkurgan Tajik Autonomous County, which is located in the eastern part of the Pamir Plateau.

The Pharmacogenetics and Pharmacogenomics Knowledge Base (PharmGKB: http://www.pharmgkb.org) is devoted to disseminating primary data and knowledge in pharmacogenetics and pharmacogenomics and has annotated genes that are important for drug response. This information is presented in the form of Very Important Pharmacogene (VIP) summaries, pathway diagrams, and curated literature [5]. It currently contains information for more than 3000 drugs, 3000 diseases, and 26,000 genes with genotyped variants [4].

We systematically genotyped 85 VIP variants selected from PharmGKB VIP in 100 Tajiks from Xinjiang [6]. We compared genotype frequencies and haplotype construction with those in Han Chinese (CHB), Japanese in Tokyo (JPT), Utah Residents with Northern and Western European Ancestry (CEU), and Yorubia in Ibadan, Nigeria (YRI). Our goals were to identify differences and determine their extent and provide a theoretical basis for safer drug administration and better therapeutic treatment in the Tajik population.

## Methods

### Ethics statement

All participants recruited and genotyped in the present study had at least three generations of paternal ancestry in their ethnic group, and each subject provided written informed consent. The Ethics Committees of Xinjiang University and Northwest University approved the use of human samples in this study.

### Study participants

We recruited a random sample of 100 healthy, unrelated Tajiks (50 males and 50 females) from Tashkurgan Tajik Autonomous County between July and October 2010 using detailed recruitment and exclusion criteria. All of the chosen subjects were Tajik Chinese living in the Xinjiang Uygur Autonomous Region.

### Polymerase chain reaction (PCR) and DNA sequencing

We successfully genotyped 85 VIP variants in 37 pharmacogenomic genes in 100 participants. Genomic DNA from whole blood was isolated using the GoldMag® nanoparticles method according to the manufacturer’s protocol, and DNA concentration was measured by spectrometry (DU530 UV/VIS spectrophotometer, Beckman Instruments, Fullerton, CA, USA). We designed primers for amplification and extension reactions using Sequenom MassARRAY Assay Design 3.0 Software [6] and used a Sequenom MassARRAY RS1000 to genotype the single nucleotide polymorphisms (SNPs) using the protocol recommended by the manufacturer. Sequenom Typer 4.0 Software was used for data management and analysis [6,7].

### Data analysis

Statistical analyses were performed using Microsoft Excel (Redmond, WA, USA) and SPSS 16.0 statistical package (SPSS, Chicago, IL, USA). All *p* values in this study were two-sided, and *p* ≤ 0.005 after Bonferroni correction was considered the statistical significance threshold [8]. We calculated and compared the genotype frequencies of Tajiks and four other populations (CHB, JPT, CEU, and YRI) using chi-squared tests [9]. We used the Haploview software package (version 4.2) for analysis of linkage disequilibrium (LD), haplotype construction, and genetic associations at polymorphic loci [10-12]. Our method excluded SNPs with minor allele frequency < 0.001 for SNPs with lower frequencies that have little power to detect LD. We also ignored SNPs with Hardy-Weinberg equilibrium (HWE) *p* values < 0.001 for their small probability that their deviation from HWE could be explained by chance. The D’ values on the square is a measure of the LD extent for each pair of SNPs, squares in red without D’ values indicate the two sites are in complete LD (D’ = 1). We constructed haplotypes using the common sites of the selected SNPs and sites downloaded from HapMap for the *VDR* gene and derived the haplotype frequencies in all five populations.

## Results

We successfully sequenced 85 VIP pharmacogenomic variant genotypes from 100 Tajiks. The PCR primers used for the selected variants are listed in Additional file 1. Table 1 lists the basic characteristics of the selected variants, including gene name, chromosome number and position, and their allele frequencies in Tajiks.

**Table 1:** **Basic characteristics of the selected variants**

**SNP ID**	**Genes**	**Chromosome**	**Position**	**Allele**		**Allele frequencies**	
				**A**	**B**	**A(%)**	**B(%)**
rs1801131	MTHFR	1	11854476	C	A	35.0	65.0
rs1801133	MTHFR	1	11856378	T	C	19.2	80.8
rs890293	CYP2J2	1	60392494	G	T	48.5	51.5
rs3918290	DPYD	1	97915614	G	/	100	0
rs6025	F5	1	169519049	C	A	100	0
rs20417	PTGS2	1	186650320	G	C	97.0	3.0
rs689466	PTGS2	1	186650750	A	G	85.4	14.7
rs4124874	UGT1A10	2	234665659	C	A	42.8	57.2
rs10929302	UGT1A10	2	234665782	G	A	73.5	26.5
rs4148323	UGT1A10	2	234669144	A	G	3.5	96.5
rs7626962	SCN5A	3	38620907	G	/	100	0
rs1805124	SCN5A	3	38645420	G	A	29.0	71.0
rs6791924	SCN5A	3	38674699	G	/	100	0
rs3814055	NR1I2	3	119500034	C	T	58.0	42.0
rs2046934	P2RY12	3	151057642	T	C	90.0	10.0
rs1065776	P2RY1	3	152553628	T	C	6.1	93.9
rs701265	P2RY1	3	152554357	G	A	20.0	80.0
rs975833	ADH1A	4	100201739	G	C	74.2	25.8
rs2066702	ADH1B	4	100229017	C	T	97.5	2.5
rs1229984	ADH1B	4	100239319	G	A	70.5	29.5
rs698	ADH1C	4	100260789	A	G	67.0	33.0
rs17244841	HMGCR	5	74607099	A	/	100	0
rs3846662	HMGCR	5	74615328	T	C	48.5	51.5
rs17238540	HMGCR	5	74619742	T	/	100	0
rs1042713	ADRB2	5	148206440	G	A	60.5	39.5
rs1042714	ADRB2	5	148206473	G	C	34.0	66.0
rs1800888	ADRB2	5	148206885	C	T	98.0	2.0
rs1142345	TPMT	6	18130918	G	A	0	100
rs1800460	TPMT	6	18139228	A	G	0	100
rs2066853	AHR	7	17379110	G	A	82.5	17.5
rs1045642	ABCB1	7	87138645	T	C	57.1	42.9
rs2032582	ABCB1	7	87160617	G	T	42.7	57.3
rs2032582	ABCB1	7	87160617	G	A	86.4	13.6
rs2032582	ABCB1	7	87160617	T	A	92.7	7.4
rs1128503	ABCB1	7	87179601	T	C	58.1	41.9
rs10264272	CYP3A5	7	99262835	C	/	100	0
rs776746	CYP3A5	7	99270539	G	A	89.5	10.5
rs4986913	CYP3A4	7	99358459	C	T	99.0	1.0
rs4986910	CYP3A4	7	99358524	T	/	100	0
rs4986909	CYP3A4	7	99359670	C	/	100	0
rs12721634	CYP3A4	7	99381661	T	/	100	0
rs2740574	CYP3A4	7	99382096	A	G	98.5	1.5
rs3815459	KCNH2	7	150644394	A	G	40.5	59.5
rs36210421	KCNH2	7	150644428	G	T	99.0	1.0
rs12720441	KCNH2	7	150647304	C	/	100	0
rs3807375	KCNH2	7	150667210	A	G	43.0	57.0
rs4986893	CYP2C19	10	96540410	G	/	100	0
rs4244285	CYP2C19	10	96541616	G	A	92.5	7.5
rs1799853	CYP2C9	10	96702047	C	T	100	0
rs1801252	ADRB1	10	115804036	G	A	20.2	79.8
rs1801253	ADRB1	10	115805055	C	G	79.8	20.2
rs5219	KCNJ11	11	17409572	C	T	56.1	43.9
rs1695	GSTP1	11	67352689	A	G	77.0	23.0
rs1138272	GSTP1	11	67353579	T	C	9.0	91.0
rs1800497	DRD2	11	113270828	T	C	17.9	82.1
rs6277	DRD2	11	113283459	G	A	61.5	38.5
rs4149056	SLCO1B1	12	21331549	T	C	90.5	9.5
rs7975232	VDR	12	48238837	C	A	49.0	51.0
rs1544410	VDR	12	48239835	G	A	66.0	34.0
rs2239185	VDR	12	48244559	T	C	51.0	49.0
rs1540339	VDR	12	48257326	G	A	67.2	32.8
rs2239179	VDR	12	48257766	A	G	56.5	43.5
rs3782905	VDR	12	48266167	C	G	70.0	30.0
rs2228570	VDR	12	48272895	T	C	34.5	65.5
rs10735810	VDR	12	48272895	C	T	66.5	33.5
rs11568820	VDR	12	48302545	G	A	77.3	22.7
rs1801030	SULT1A2	16	28617485	A	/	100	0
rs3760091	SULT1A1	16	28620800	C	G	54.1	45.9
rs7294	VKORC1	16	31102321	C	T	67.0	33.0
rs9934438	VKORC1	16	31104878	G	A	50.5	49.5
rs28399454	CYP2A6	19	41351267	G	/	100	0
rs28399444	CYP2A6	19	41354190	A	/	100	0
rs1801272	CYP2A6	19	41354533	T	/	100	0
rs28399433	CYP2A6	19	41356379	G	T	10.5	89.5
rs3745274	CYP2B6	19	41512841	G	T	64.0	36.0
rs28399499	CYP2B6	19	41518221	T	/	100	0
rs3211371	CYP2B6	19	41522715	C	T	50.0	50.0
rs12659	SLC19A1	21	46951555	C	T	56.6	43.4
rs1051266	SLC19A1	21	46957794	G	A	55.7	44.3
rs1131596	SLC19A1	21	46957915	T	C	60.6	39.4
rs4680	COMT	22	19951271	A	G	53.5	46.5
rs59421388	CYP2D6	22	42523610	C	/	100	0
rs28371725	CYP2D6	22	42523805	G	A	90.0	10.0
rs16947	CYP2D6	22	42523943	G	A	74.1	25.9
rs5030656	CYP2D6	22	42524175	AAG	delAAG	99.5	0.5
rs61736512	CYP2D6	22	42525134	C	/	100	0
rs28371706	CYP2D6	22	42525772	C	T	99.0	1.0

Table 2 lists the genotype frequencies in Tajiks and identifies significant variants in Tajiks compared with the other four populations (*p* < 0.005), all variant data are shown in Additional file 2. We also categorized the genes into different families and phases related to pharmacogenomics, the statistically significant values are shown in red (*p* < 0.05). We found that Tajiks differed from CHB, JPT, and YRI in 30, 32, and 32 selected VIP genotypes, respectively. These genes encode phase I drug metabolic enzymes (*VCORC1, MTHFR,* and *CYP3A5*), a phase II drug metabolic enzymes (*COMT*)*,* and transporters, channel proteins, and receptors (e.g., *ADRB1*, *KCNH2,* and *VDR*, respectively). However, the difference between Tajiks and CEU was much smaller; just six SNP genotypes were different, and these were randomly distributed on genes such as *CYP2C9*, which encodes a phase I enzyme. For genes such as *ADH1B* and *PTGS2*, we observed differences between Tajiks and the other four populations.

**Table 2:** **Genotype frequencies in Tajiks compared with four other populations**

**SNP ID**	**Gene**	**Category**		**Allele**		**Tajik genotype frequencies**			***p*** **values against four populations (after Bonferroni correction)**			
		**Family**	**Phase**	**A**	**B**	**AA(%)**	**AB(%)**	**BB(%)**	**CHB**	**JPT**	**CEU**	**YRI**
rs1045642	ABCB1	ABC transporters	others	T	C	34.3	45.5	20.2	8.49E-03	3.42E-02	3.10E-01	2.17E-18
rs1128503	ABCB1	ABC transporters	others	T	C	32.3	51.5	16.2	3.09E-02	8.73E-01	1.69E-02	3.03E-19
rs2032582	ABCB1	ABC transporters	others	G	T	18	49.4	32.6	9.02E-01	6.07E-01	8.24E-02	-
rs975833	ADH1A	alcohol dehydrogenase	phase I	G	C	55.6	37.4	7.1	4.86E-16	3.45E-14	6.46E-01	5.79E-01
rs1229984	ADH1B	alcohol dehydrogenase	phase I	G	A	48	45	7	3.76E-12	7.23E-11	9.18E-11	1.26E-10
rs2066702	ADH1B	alcohol dehydrogenase	phase I	C	T	95	5.1	0	2.97E-01	2.97E-01	1.94E-01	1.61E-11
rs698	ADH1C	alcohol dehydrogenase	phase I	A	G	44.3	45.4	10.3	1.21E-09	3.22E-09	1.24E-02	7.40E-11
rs1801252	ADRB1	adrenergic receptors	others	G	A	3	34.3	62.6	1.22E-05	1.52E-05	-	2.53E-06
rs1801253	ADRB1	adrenergic receptors	others	C	G	63.6	32.3	4	6.12E-01	3.88E-01	5.87E-02	4.12E-04
rs1042713	ADRB2	adrenergic receptors	others	G	A	36	49	15	4.49E-03	5.67E-01	7.34E-01	6.05E-02
rs1042714	ADRB2	adrenergic receptors	others	G	C	14	40	46	1.26E-03	3.39E-05	2.70E-02	9.85E-03
rs2066853	AHR	AHR	others	G	A	69	27	4	1.18E-05	7.65E-08	6.87E-02	9.06E-08
rs4680	COMT	COMT	phase II	A	G	31	45	24	9.95E-05	1.56E-05	5.25E-01	3.15E-05
rs28399454	CYP2A6	cytochrome P450	phase I	G	/	100	0	0	-	-	1.00E + 00	7.81E-07
rs3745274	CYP2B6	cytochrome P450	phase I	G	T	46	36	18	1.07E-03	2.03E-03	5.17E-02	1.50E-01
rs28399499	CYP2B6	cytochrome P450	phase I	T	/	100	0	0	-	-	-	2.04E-06
rs4244285	CYP2C19	cytochrome P450	phase I	G	A	85	15	0	1.00E-03	1.56E-05	4.07E-02	8.50E-02
rs1799853	CYP2C9	cytochrome P450	phase I	C	T	100	0	0	-	-	6.25E-05	-
rs776746	CYP3A5	cytochrome P450	phase I	G	A	81	17	2	2.78E-05	8.74E-05	2.58E-02	1.23E-34
rs10264272	CYP3A5	cytochrome P450	phase I	C	/	100	0	0	1.00E + 00	1.00E + 00	-	8.95E-09
rs6277	DRD2	G-protein-coupled receptor	others	G	A	38	47	15	7.41E-08	1.01E-07	1.98E-02	6.51E-11
rs1800497	DRD2	G-protein-coupled receptor	others	T	C	3.1	29.6	67.4	8.59E-06	1.43E-05	7.19E-01	2.25E-06
rs1695	GSTP1	glutathione S-transferase	phase II	A	G	59	36	5	5.70E-01	1.96E-03	2.32E-04	1.50E-03
rs1138272	GSTP1	glutathione S-transferase	phase II	T	C	0	18	82	3.00E-03	2.00E-03	9.22E-01	2.28E-03
rs3846662	HMGCR	HMGCR	phase I	T	C	18	61	21	2.49E-01	7.26E-01	2.12E-02	2.79E-24
rs3807375	KCNH2	eag	others	A	G	19	48	33	6.73E-07	8.34E-12	4.02E-01	1.13E-11
rs3815459	KCNH2	eag	others	A	G	16	49	35	6.04E-06	2.69E-09	-	6.88E-01
rs1801131	MTHFR	methylenetetrahydrofolate reductase	phase I	C	A	7	56	37	9.07E-03	5.46E-04	2.32E-01	7.36E-09
rs1801133	MTHFR	methylenetetrahydrofolate reductase	phase I	T	C	4	30.3	65.7	3.13E-07	1.21E-03	1.92E-02	9.34E-03
rs3814055	NR1I2	nuclear receptor	others	C	T	30	56	14	8.60E-03	2.15E-03	1.00E-01	1.32E-03
rs701265	P2RY1	G-protein coupled receptor	others	G	A	4	32	64	7.61E-02	3.04E-01	9.33E-01	2.96E-25
rs2046934	P2RY12	G-protein coupled receptor	others	T	C	82	16	2	5.69E-02	4.97E-02	4.49E-03	6.23E-03
rs20417	PTGS2	nuclear receptor	others	G	C	97	0	3	5.43E-04	1.57E-03	3.65E-07	4.82E-17
rs689466	PTGS2	nuclear receptor	others	A	G	73.7	23.2	3	3.68E-11	4.34E-07	7.13E-01	1.12E-01
rs1805124	SCN5A	sodium channel gene	others	G	A	9	40	51	1.23E-04	7.59E-04	7.80E-03	4.96E-01
rs6791924	SCN5A	sodium channel gene	others	G	/	100	0	0	-	-	-	2.00E-03
rs7626962	SCN5A	sodium channel gene	others	G	/	100	0	0	-	-	-	1.24E-03
rs1051266	SLC19A1	solute carrier	others	G	A	29.9	51.6	18.6	3.65E-01	8.28E-02	8.36E-01	2.83E-06
rs4149056	SLCO1B1	solute carrier	others	T	C	82	17	1	2.30E-01	7.68E-01	1.47E-01	2.66E-04
rs4124874	UGT1A10	UDP-glucuronosyltransferase	phase II	C	A	14.4	56.7	28.9	1.64E-02	6.88E-02	3.31E-01	2.73E-23
rs4148323	UGT1A10	UDP-glucuronosyltransferase	phase II	A	G	0	7	93	7.23E-08	3.22E-03	9.00E-02	9.00E-02
rs10929302	UGT1A10	UDP-glucuronosyltransferase	phase II	G	A	53	41	6	2.58E-03	3.31E-03	9.38E-01	4.91E-02
rs1540339	VDR	nuclear receptor	others	G	A	47.5	39.4	13.1	1.60E-10	1.42E-11	7.78E-01	1.80E-02
rs1544410	VDR	nuclear receptor	others	G	A	40	52	8	3.05E-12	2.03E-06	1.75E-02	2.50E-01
rs2239179	VDR	nuclear receptor	others	A	G	31	51	18	4.01E-04	6.14E-05	3.63E-02	8.58E-03
rs2239185	VDR	nuclear receptor	others	T	C	22	58	20	2.86E-04	1.55E-01	-	4.37E-01
rs3782905	VDR	nuclear receptor	others	C	G	46	48	6	1.20E-01	6.72E-04	3.92E-01	7.74E-02
rs7975232	VDR	nuclear receptor	others	C	A	20	58	22	9.30E-05	2.13E-03	1.92E-02	2.44E-02
rs10735810	VDR	nuclear receptor	others	C	T	48.4	36.3	15.4	8.17E-02	8.74E-01	2.72E-01	4.64E-03
rs11568820	VDR	nuclear receptor	others	G	A	60.6	33.3	6.1	1.12E-04	4.25E-05	6.86E-01	8.35E-38
rs7294	VKORC1	VKORC1	phase I	C	T	44	46	10	8.00E-10	1.09E-06	7.69E-01	1.59E-04
rs9934438	VKORC1	VKORC1	phase I	G	A	26	49	25	1.29E-17	4.64E-14	1.01E-01	9.39E-25

We counted the variants in each family, excluding those that belonged to none of the families or were not significantly different between Tajiks and the other four populations. The remaining 71 sites belonged to 26 genes in 12 families (Table 3). We found that the difference between Tajiks and CEU existed in only one site in the nuclear receptor family and 0 site in adrenergic receptors family respectively. However, in the nuclear receptor family, Tajiks differed from CHB, JPT, and YRI in 66.7%, 75%, and 33.3% of selected sites, respectively. In the adrenergic receptor family, Tajiks differed from CHB, JPT, and YRI in 60%, 40%, and 40% of selected sites, respectively. For genes in ATP-binding cassette (ABC) transporters, Tajiks differed from YRI in 66.7% of the selected sites, but there was no difference between Tajiks and CHB, JPT, CEU.

**Table 3:** **Numbers and frequencies of significant variants**

**Family**	**Variants (n)**	**Significant variants, n (%)**			
		**CHB**	**JPT**	**CEU**	**YRI**
Adrenergic receptors	5	3 (60.0)	2 (40.0)	0 (0)	2 (40.0)
Alcohol dehydrogenase	4	3 (75.0)	3 (75.0)	1 (25.0)	3 (75.0)
ATP-binding cassette (ABC) transporters	3	0 (0)	0 (0)	0 (0)	2 (66.7)
Cytochrome P450	24	3 (24.9)	3 (24.9)	1 (8.3)	4 (16.7)
Eag	4	2 (50.0)	2 (50.0)	0 (0)	1 (25.0)
Glutathione S-transferase	2	1 (50.0)	2 (100)	1 (50.0)	2 (100)
G-protein coupled receptor	5	2 (40.0)	2 (40.0)	1 (20.0)	3 (60.0)
Methylenetetrahydrofolate reductase	2	1 (50.0)	2 (100.0)	0 (0)	1 (50.0)
Nuclear receptor	12	8 (66.7)	9 (75.0)	1 (8.3) 0)	4 (33.3)
Sodium channel gene	3	1 (33.3)	1 (33.3)	0 (0)	2 (66.7)
Solute carrier	4	0 (0)	0 (0)	0 (0)	2 (50.0)
UDP-glucuronosyltransferase	3	2 (33.7)	2 (66.7)	0 (0)	1 (33.3)

**Figure 1:**
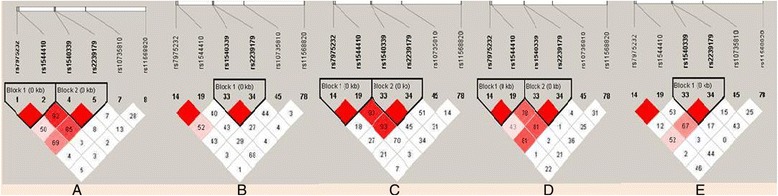
**Linkage disequilibrium (LD) analysis of** ***VDR*** **in five populations.** LD is displayed by standard color schemes, with bright red for very strong LD (LOD > 2, D’ = 1), light red (LOD > 2, D’ < 1) and blue (LOD < 2, D’ = 1) for intermediate LD, and white (LOD < 2, D’ < 1) for no LD. **A**. Tajiks, **B**. CHB, **C**. JPT, **D**. CEU, **E**. YRI.

We performed LD analysis using Haploview to define blocks and haplotypes. Using the common sites of our study and those from HapMap in the *VDR* gene, we identified two LD blocks in Tajiks, JPT, and CEU and one LD block in CHB and YRI (Figure 1). The block identified in all five populations spans 0.4 kb and consists of two complete LD markers (rs1540339 and rs2239179) with a D’ value equal to 1. The block identified in Tajiks, JPT, and CEU spans 0.9 kb and also consists of two complete LD markers (rs7975232 and rs1544410) with a D’ value equal to 1.

**Figure 2:**
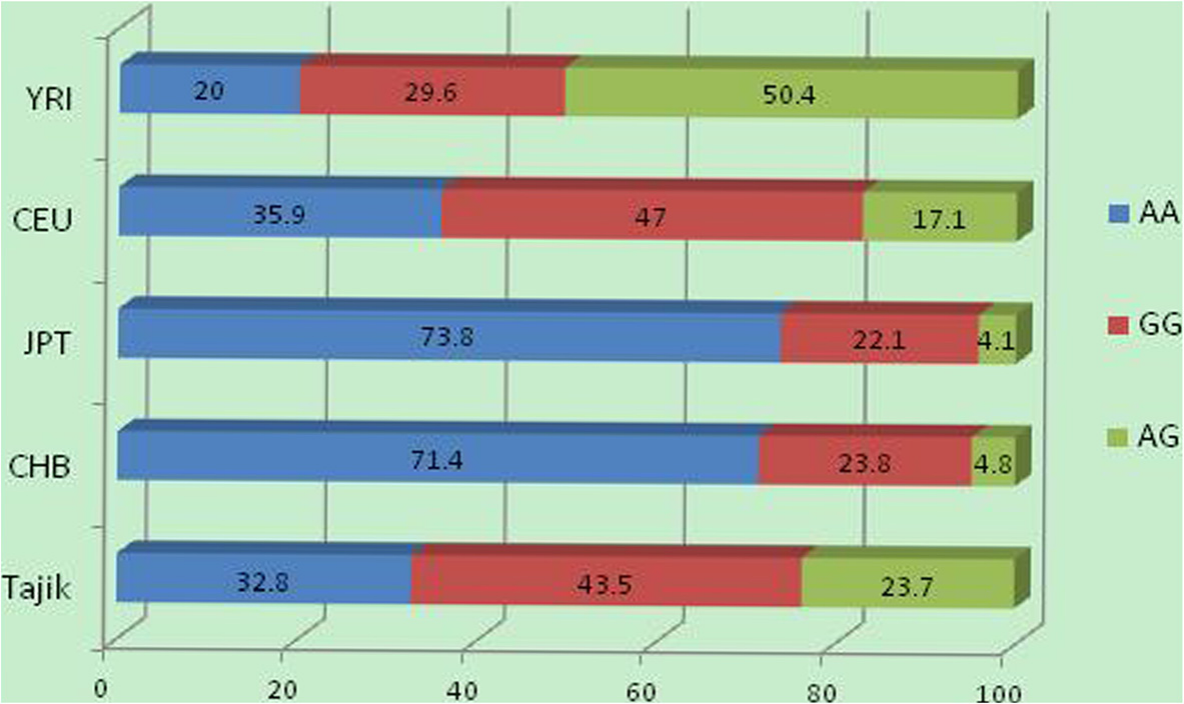
**Haplotype analysis results of rs1540339 and rs2239179 in** ***VDR*** **.**

Haplotype analysis results are shown in Figure 2. For the common block comprised of rs1540339 and rs2239179, three kinds of haplotypes were identified in all five populations, but they differed in frequency. Three colors of bars indicate the three kinds of haplotypes. The highest and lowest frequencies of haplotype “AA” were found in JPT (73.8%) and YRI (20.0%). The highest and lowest frequencies of haplotype “GG” were observed in CEU (47.0%) and JPT (22.1%). The highest and lowest frequencies of haplotype “GA” were found in YRI (50.4%) and JPT (4.1%). The haplotype constitutions and frequencies show that there are relatively minimal differences between Tajik and CEU, CHB, and JPT, whereas the differences between YRI and the other four populations seem obvious. These findings are in accordance with the results shown in Table 3.

## Discussion

With the rapid development of pharmacogenetics, serious attention has been given to interethnic and interracial differences in drug responses [13]. Here, we genotyped 85 variants related to pharmacogenomics in the Tajik ethnic group for the first time and compared the results with other ethnic populations around the world. We found that 30, 32, 32, and 6 VIP variants differed from CHB, JPT, YRI, and CEU respectively (*p* < 0.005). These findings corroborate the current opinion that polymorphisms with varying frequencies occur among different populations.

Vitamin D receptor (*VDR*) is a gene whose function has been widely reported. Epithelial cells convert the primary circulating form of vitamin D to its active form, which binds VDR to regulate a variety of genes that keep cellular proliferation and differentiation within normal ranges to prevent malignant transformation [14]. That is to say, the active form of vitamin D can induce apoptosis and prevent angiogenesis by binding *VDR*, which reduces the survival potential of malignant cells. Studies have demonstrated that rs10735810 and rs1544410 SNPs in *VDR* might modulate the risk of breast, skin, and prostate cancers, as well as other forms [15,16]. An Italian study reported that GA and AA rs1544410 genotypes were associated with decreased cutaneous malignant melanoma (CMM) risk (odds ratio = 0.78 and 0.75, respectively) compared with the GG genotype [16]. A study in Japan found that head and neck squamous cell carcinoma patients with the TT rs10735810 genotype was associated with poor progression-free survival compared with CC or CT genotype patients (log-rank test, *p* = 0.0004; adjusted hazard ratio, 3.03; 95% confidence interval, 1.62 to 5.67; *p* = 0.001), and the A-T-G (rs11568820-rs10735810-rs7976091) haplotype showed a significant association with a higher progression rate (*p* = 0.02). [14] We found that the GA and AA genotype frequencies of rs1544410 in Tajiks were as much as 52% and 8% respectively, which is different from those in CHB and JPT (data not shown), suggesting that Tajiks may have decreased susceptibility to CMM.

The gene alcohol dehydrogenase 1B (*ADH1B*) produces a key protein for alcohol metabolism that determines blood acetaldehyde concentrations after drinking [17]. This member of the alcohol dehydrogenase family also metabolizes a wide variety of substrates besides ethanol, including retinol, other aliphatic alcohols, hydroxysteroids, and lipid peroxidation products. The minor allele “A” of rs1229984 encodes a super-active allozyme that is reportedly associated with lower rates of alcohol dependence in numerous association studies, and its frequency varies widely across different populations. It is 69% (19-91%) in normal Asian normal populations, 5.5% (1-43%) in normal European populations, and just 3% (2-7%) in normal Mexican populations [18]. Other studies have shown that rs1229984 may influence alcohol consumption behavior and is associated with upper aerodigestive (UADT) cancers [19-24]. A genome-wide association study found that the “A” allele of rs1229984 was associated with decreased UADT risk (*p* = 7 × 10^−9^) [19]. The data in our study is in accordance with previous findings; we found that the “A” allele frequency of rs1544410 in Tajiks was 29.5%, which was significantly different (*p* < 0.05) from 76.67%, 73.86%, 0%, and 0% in CHB, JPT, CEU, and YRI respectively, suggesting that Tajiks have an intermediate susceptibility to UADT cancer.

The catechol-o-methyltransferase gene (*COMT*) is responsible for eliminating dopamine from the synaptic cleft in the prefrontal cortex (PFC) [25]. Variations in the *COMT* gene exert complex effects on susceptibility to depression through various intermediate phenotypes, such as impulsivity and executive function [26]. The common functional *COMT* polymorphism rs4680 has been shown to affect enzyme activity and, consequently, intrasynaptic dopamine content. The “G” allele is associated with 40% higher enzymatic activity in the human brain compared to the “A” allele, leading to more efficient elimination of dopamine from the synaptic cleft; therefore, the GG genotype is associated with reduced synaptic dopamine in the PFC, and in turn, more active striatal dopamine neurotransmission [25,27-29]. A study in northern Italy reported an association between the GG genotype and the risks of Alzheimer’s disease (AD) and its precursor, mild cognitive impairment (MCI) [30]. The GG genotype frequency in our study was just 24% in Tajiks, compared with 51.2%, 50%, and 46% in CHB, JPT, and YRI respectively (*p* < 0.05). This suggests that Tajiks may be less vulnerable to diseases related to dopamine content, including AD and MCI.

Our study also found significant differences in genotype frequencies between Tajiks and other populations in genes such as *DRD2* and *F5*. Polymorphisms in these genes have been shown to be associated with dyskinesia induced by levodopa therapy in Parkinson’s disease patients and coronary artery disease, respectively [31,32].

The Tajiks speak a western Indo-Iranian language and their presence in China dates to the 10^th^-century Muslim invasion, suggesting they are descendants of eastern Indo-Iranian speakers [33]. This may explain the smaller differences between Tajiks and CEU compared to other three populations we investigated.

However, intrinsic limitations still exist in our study. Our sample size is relatively not big enough, thus further investigation related to pharmacogenomics gene polymorphisms in a larger Tajik population is necessary to ascertain the results obtained in the current study.

## Conclusions

These results provide the first pharmacogenomics information in Tajiks and illustrate the difference of selected genes between Tajiks and four other populations. Present-day China is a nation with 56 distinct ethnic groups. Our study provides a theoretical basis for safer drug administration and better therapeutic treatments in this unique population, and may also be applied in the diagnosis and prognosis of specific diseases in Tajiks.

## Additional files

Additional file 1:
**PCR primers for the selected variants.**
Additional file 2:
**Genotype frequencies in Tajiks compared with four other populations.**
