# Supplementary material for: Genetic polymorphisms of VIP variants in the Tajik ethnic group of northwest China
Source: BMC Genet. 2014 Sep 30;15:102. doi: 10.1186/s12863-014-0102-y (PMC4189671; doi:10.1186/s12863-014-0102-y)
Supplement: Additional file 1: — PCR primers for the selected variants. [file 12863_2014_102_MOESM1_ESM.doc]

| **Additional file 1 PCR primers for the selected variants** | | | | |
| --- | --- | --- | --- | --- |
| SNP_ID | Gene | Forward primer (5'-3') | Reverse primer (5'-3') | Primer for extension reactions |
| rs4124874 | UGT1A10 | ACGTTGGATGGTCATAGTAAGCTGGCCAAG | ACGTTGGATGTCCCCAAACTTCCTTTGATG | ccccTGTTCTCAAATTGCTTTGTTCA |
| rs10929302 | UGT1A10 | ACGTTGGATGTAGAGGGTAAGAGGCAGAGG | ACGTTGGATGACTCTGGGATAGACCCCAGC | CCCCAGCCCACCTGTC |
| rs1051266 | SLC19A1 | ACGTTGGATGAGAAGCAGGTGCCCGTGGAA | ACGTTGGATGCGTAGAAGCAAAGGTAGCAC | AAAGGTAGCACACGAGG |
| rs1042713 | ADRB2 | ACGTTGGATGGAACGGCAGCGCCTTCTTG | ACGTTGGATGACCCACACCTCGTCCCTTT | gCCGGCGCATGGCTTC |
| rs1229984 | ADH1B | ACGTTGGATGCTGAATCTGAACAGCTTCTC | ACGTTGGATGTTGCCACTAACCACGTGGTC | cctcACCACGTGGTCATCTGTG |
| rs701265 | P2RY1 | ACGTTGGATGCTCCTCTGAGGAGAAAATCG | ACGTTGGATGCATGGAAAGGGATGTAAGAC | tagaAAAAACAGTCAGTACAATGAT |
| rs4986893 | CYP2C19 | ACGTTGGATGAACATCAGGATTGTAAGCAC | ACGTTGGATGGACTGTAAGTGGTTTCTCAG | tACTTGGCCTTACCTGGAT |
| rs4244285 | CYP2C19 | ACGTTGGATGGCAATAATTTTCCCACTATC | ACGTTGGATGCACTTTCCATAAAAGCAAGG | AGTAATTTGTTATGGGTTCC |
| rs16947 | CYP2D6 | ACGTTGGATGTCACCATCCCGGCAGAGAA | ACGTTGGATGCCCTGAGAGCAGCTTCAATG | gaatCTTCAATGATGAGAACCTG |
| rs11568820 | VDR | ACGTTGGATGCATCTTTTGTATCAGGAAC | ACGTTGGATGAACTGCAACCCATAATAAG | ACCCATAATAAGAAATAAGTTTTTA |
| rs7294 | VKORC1 | ACGTTGGATGTAGATTACCCCCTCCTCCTG | ACGTTGGATGAAAAAAGAGCGAGCGTGTGG | TGGTCCATTGTCATGTG |
| rs28371725 | CYP2D6 | ACGTTGGATGGAGCCCATCTGGGAAACAGT | ACGTTGGATGTCCCAGCAAAGTTCATGGGC | gaCCCGCCTGTACCCTT |
| rs1800460 | TPMT | ACGTTGGATGACTTACCATTTGCGATCACC | ACGTTGGATGGCAAATTTGACATGATTTGGG | CATGATTTGGGATAGAGGA |
| rs6025 | F5 | ACGTTGGATGAATCTGTAAGAGCAGATCCC | ACGTTGGATGCTGAAAGGTTACTTCAAGGAC | GACAAAATACCTGTATTCCT |
| rs4148323 | UGT1A10 | ACGTTGGATGGCACAGGGTACGTCTTCAAG | ACGTTGGATGAGTTGTCCTAGCACCTGACG | ccccaCTCGTTGTACATCAGAGAC |
| rs776746 | CYP3A5 | ACGTTGGATGCCATAATCTCTTTAAAGAGC | ACGTTGGATGGATGAAGGGTAATGTGGTCC | aatggGGTCCAAACAGGGAAGAGATA |
| rs1544410 | VDR | ACGTTGGATGTAGATAAGCAGGGTTCCTGG | ACGTTGGATGAGCCCAGTTCACGCAAGAG | GCCTGAGTATTGGGAATG |
| rs2032582 | ABCB1 | ACGTTGGATGGAAAATGTTGTCTGGACAAGC | ACGTTGGATGCATATTTAGTTTGACTCACC | ccagTTGACTCACCTTCCCAG |
| rs1801131 | MTHFR | ACGTTGGATGTCTCCCGAGAGGTAAAGAAC | ACGTTGGATGAGGAGCTGCTGAAGATGTGG | ccccaGAGCTGACCAGTGAAG |
| rs7975232 | VDR | ACGTTGGATGTAGAGAAGAAGGCACAGGAG | ACGTTGGATGTTGAGTGTCTGTGTGGGTGG | GGATTGAGCAGTGAGG |
| rs975833 | ADH1A | ACGTTGGATGATATATTAAGTCCAGTTCC | ACGTTGGATGTTAACCAACTCACAGATTGC | ATTATGTGATGATGAAATTGTATG |
| rs1042714 | ADRB2 | ACGTTGGATGGAAGCCATGCGCCGGACCA | ACGTTGGATGAGACATGACGATGCCCATGC | ACACCTCGTCCCTTT |
| rs20417 | PTGS2 | ACGTTGGATGAGGACCAGTATTATGAGGAG | ACGTTGGATGTGTTCTCCGTACCTTCACCC | TGTTTCTTGGAAAGAGAGG |
| rs2228570 | VDR | ACGTTGGATGTGGCCTGCTTGCTGTTCTTA | ACGTTGGATGAAGTCTCCAGGGTCAGGCA | cctgGCTGGCCGCCATTGCCTCC |
| rs2239185 | VDR | ACGTTGGATGACAGCAACACAATTCCAGTC | ACGTTGGATGGTGACATTTACACCCTCCTC | CCCTCCTCTGTCTTCAC |
| rs1045642 | ABCB1 | ACGTTGGATGTATGGAGACAACAGCCGGGT | ACGTTGGATGAAGGCATGTATGTTGGCCTC | ctaCTTTGCTGCCCTCAC |
| rs5219 | KCNJ11 | ACGTTGGATGTGACACGCCTGGCAGAGGA | ACGTTGGATGCCTTTCTTGGACACAAAGCG | ggaGGGCACGGTACCTGGGCT |
| rs4149056 | SLCO1B1 | ACGTTGGATGGAATCTGGGTCATACATGTG | ACGTTGGATGTATGGGAGTCTCCCCTATTC | tcaACGAAGCATATTACCCATGAAC |
| rs3846662 | HMGCR | ACGTTGGATGCCTTAAAACTCTTCTCATTG | ACGTTGGATGGCAATAGGTGTAAGTTGGC | gatgTGCCAGTTTAAAAATACATCATA |
| rs28399499 | CYP2B6 | ACGTTGGATGTCTGTACAGAGAGAGTCTAC | ACGTTGGATGTTGGCTCGGTCATGAAGCTC | cGCCAATCACCTGTTCA |
| rs1128503 | ABCB1 | ACGTTGGATGGTTTTTTTCTCACTCGTCCTG | ACGTTGGATGTCTGCCCACTCTGCACCTT | caaTCTGCACCTTCAGGTTCAG |
| rs2046934 | P2RY12 | ACGTTGGATGCAATTTCACTTATCTCTGG | ACGTTGGATGTATGGCATCTACATCTTGGG | ggaggTCTTGGGAATTTGAAATGAC |
| rs1131596 | SLC19A1 | ACGTTGGATGTTCCAAGGTGCCCTGACTC | ACGTTGGATGCTCAGGCCACGTGCAGCTC | gggtGAGGGGACGAAGGTGAC |
| rs1695 | GSTP1 | ACGTTGGATGTGGTGCAGATGCTCACATAG | ACGTTGGATGATGGTGAATGACGGCGTGGA | cagtACCTCCGCTGCAAATAC |
| rs1540339 | VDR | ACGTTGGATGACACACATTCTCAGTGGGC | ACGTTGGATGGTGCTGAGCACACCTTGTTG | TGTTGGTGCCCACCCTAA |
| rs2066853 | AHR | ACGTTGGATGCCTAGGCATTGATTTTGAAG | ACGTTGGATGCACCAGAAAAATCATTTCTGA | TTTTTCATTCTGCATGTGT |
| rs689466 | PTGS2 | ACGTTGGATGATTAGATGGAAGGGAGATT | ACGTTGGATGCCTGAGCACTACCCATGATA | CAAAAGCAAAGATGAAATTCCA |
| rs1805124 | SCN5A | ACGTTGGATGATGATGAAAACAGCACAGCG | ACGTTGGATGGGGCCAGGGCACCAGCAGT | aaagtGCACCAGCAGTGATGTG |
| rs1142345 | TPMT | ACGTTGGATGGTTGGGGAATTGACTGTCTT | ACGTTGGATGCTATGTCTCATTTACTTTTC | CTCATTTACTTTTCTGTAAGTAGA |
| rs1801252 | ADRB1 | ACGTTGGATGGTCGCCGCCCGCCTCGTT | ACGTTGGATGTGTCCACTGCTGAGACAGCG | ACAGCGGCTCGGGGC |
| rs9934438 | VKORC1 | ACGTTGGATGTTCCCCGACCTCCCATCCTA | ACGTTGGATGACATGGAATCCTGACGTGGC | GCCAGGAGATCATCGAC |
| rs2740574 | CYP3A4 | ACGTTGGATGGAAACTCAAGTGGAGCCATT | ACGTTGGATGGAATGAGGACAGCCATAGAG | acatCCATAGAGACAAGGGCA |
| rs2239179 | VDR | ACGTTGGATGACTTATCCTCTGTCCCTGAC | ACGTTGGATGCTATGGGACCGTTTGGAGTG | cggTTGGAGTGGTTGGGG |
| rs3815459 | KCNH2 | ACGTTGGATGTACCAGACAACACCGCCAG | ACGTTGGATGTCAACAGGTGAGGGAGTGC | ggtGAGTGCAGGTGGGGT |
| rs698 | ADH1C | ACGTTGGATGAGAGCGAAGCAGGTCAAATC | ACGTTGGATGAAGAAGTTTTCACTGGATGC | gtgTTCACTGGATGCATTAATAACAAAT |
| rs3807375 | KCNH2 | ACGTTGGATGGGCAATGAGAGAGAACGTGT | ACGTTGGATGTCCTGAAACTCCTCCCTTAG | actaCCTTAGAGAACTTCTGCGTTTAGA |
| rs4680 | COMT | ACGTTGGATGTTTTCCAGGTCTGACAACGG | ACGTTGGATGACCCAGCGGATGGTGGATTT | ggggaGTGGATTTCGCTGGC |
| rs3782905 | VDR | ACGTTGGATGAAAGAGGCAGTGGGAGGGAG | ACGTTGGATGGGGGTCTCAAATTCTTAATG | aatgTAAAATCTACTTTCACCCACT |
| rs3760091 | SULT1A1 | ACGTTGGATGCCTAGGGTCTGGGATTATAG | ACGTTGGATGCAAAACTCTGATGACTCAGC | ATGACTCAGCAAAAGCA |
| rs1801253 | ADRB1 | ACGTTGGATGAGCCCTGCGCGCGCAGCAGA | ACGTTGGATGCCTTCAACCCCATCATCTAC | CGCAAGGCCTTCCAG |
| rs28399433 | CYP2A6 | ACGTTGGATGTGGGATGATAGATGGTGACG | ACGTTGGATGGTAATCAGCCAAAGTCCATC | aagaaATCCCTCTTTTTCAGGCAGTA |
| rs1138272 | GSTP1 | ACGTTGGATGTGATACATGGTGGTGTCTGG | ACGTTGGATGTCAAAAGGCTTCAGTTGCCC | ATAGTCATCCTTGCCC |
| rs1800497 | DRD2 | ACGTTGGATGTGTGCAGCTCACTCCATCCT | ACGTTGGATGTCAAGGGCAACACAGCCATC | aaagCCTCAAAGTGCTGGTC |
| rs1800888 | ADRB2 | ACGTTGGATGTACCAGTGCATCTGAATGGG | ACGTTGGATGTAAGGCCCGGGTGATCATTC | gggaGATTGTGTCAGGCCTTA |
| rs1801133 | MTHFR | ACGTTGGATGACCTGAAGCACTTGAAGGAG | ACGTTGGATGGAAAAGCTGCGTGATGATG | gGCGTGATGATGAAATCG |
| rs1065776 | P2RY1 | ACGTTGGATGAATGACCGAGGTGCTGTGGC | ACGTTGGATGTGCTGTTCCCCCAGGACGAA | ggaaCAGGACGAACCCGGACC |
| rs12659 | SLC19A1 | ACGTTGGATGACTGAGTCCCCACAGGCCA | ACGTTGGATGCTTCGGAGCTGGAGCGCAT | TGGAGCGCATGAATCC |
| rs3814055 | NR1I2 | ACGTTGGATGGAGACCACGATTGAGCAAAC | ACGTTGGATGTCACCTGAAGACAACTGTGG | cccgtTCATTTTTTGGCAATCCCAGGTT |
| rs6277 | DRD2 | ACGTTGGATGATTCTTCTCTGGTTTGGCGG | ACGTTGGATGACCACCAGCTGACTCTCCC | TCTCCACAGCACTCC |
| rs3211371 | CYP2B6 | ACGTTGGATGGACACTGAATGACCCTGGAA | ACGTTGGATGGCAAAATACCCCCAACATAC | gCCCCAACATACCAGATC |
| rs10735810 | VDR | ACGTTGGATGAAGTCTCCAGGGTCAGGCA | ACGTTGGATGTGGCCTGCTTGCTGTTCTTA | ccgGCTTGCTGTTCTTACAGGGA |
| rs3745274 | CYP2B6 | ACGTTGGATGTTCTTCCTAGGGGCCCTCAT | ACGTTGGATGCAAAGACGATGGAGCAGATG | GTTGGCGGTAATGGA |
| rs890293 | CYP2J2 | ACGTTGGATGTCCCAGCAGGCGACGGTCC | ACGTTGGATGTTCGCAGGGTGCTGCGAAGG | tcgtGGCACGGCTGGGAGC |
| rs28399444 | CYP2A6 | ACGTTGGATGACTGGAAGATTCCTAGCATC | ACGTTGGATGTCTTTGGGGACCGCTTTGAC | ccaCGCTTTGACTATAAGGACA |
| rs1801030 | SULT1A2 | ACGTTGGATGAAGATCCTGGAGTTTGTGGG | ACGTTGGATGATCTCCTTGAACGACGTGTG | ACGTGTGCTGAACCA |
| rs17244841 | HMGCR | ACGTTGGATGGGGAAACAAAGTGAGACTAC | ACGTTGGATGCAGGTATTCAAGATACAAAG | AAGTATGATTGTAATATAAAGGATTT |
| rs5030656 | CYP2D6 | ACGTTGGATGACTGAGGCCTTCCTGGCAGA | ACGTTGGATGACGCTCAACCCACCACCCTT | GTGGCAGCCACTCTCACCT |
| rs61736512 | CYP2D6 | ACGTTGGATGACTGCTCCAGCGACTTCTTG | ACGTTGGATGTATGGGCCCGCGTGGCGCGA | ctCAGAGGCGCTTCTCC |
| rs12720441 | KCNH2 | ACGTTGGATGTCGCCCCGCAGGATCTCGAT | ACGTTGGATGCACACTGGTGCATGCTGGG | CCGCCCTGTACTTCATCTCC |
| rs4986909 | CYP3A4 | ACGTTGGATGAGGGAGGGCTCCCTTCCCA | ACGTTGGATGCGTGACCCAAAGTACTGGAC | cttGACAGAGCCTGAGAAGTTCCTCC |
| rs10264272 | CYP3A5 | ACGTTGGATGGGATCTAAGAAACCAAATT | ACGTTGGATGCGACTCTCTCAACAATCCAC | aTTTGTGGAGAGCACTAA |
| rs1799853 | CYP2C9 | ACGTTGGATGACCCACCCTTGGTTTTTCTC | ACGTTGGATGCTGCGGAATTTTGGGATGGG | agGAGGAGCATTGAGGAC |
| rs4986913 | CYP3A4 | ACGTTGGATGAGAAAATTGACTAACCTGTG | ACGTTGGATGTGCTCTAATCAGAGTCCTTC | TTCAGAACTTCTCCTTCAAA |
| rs2066702 | ADH1B | ACGTTGGATGCTCTATTGCCTCAAAACGTC | ACGTTGGATGGCATGTGGGTTGTCTAAATG | gctaTTCTTTCCTATTGCAGTATC |
| rs59421388 | CYP2D6 | ACGTTGGATGATTGTGGGGACGCATGTCTG | ACGTTGGATGTGGTCACCCATCTCTGGTC | ataTCTGGTCGCCGCACCTGCCCTATCA |
| rs28371706 | CYP2D6 | ACGTTGGATGGCTGCTTGCCTTGGGAACG | ACGTTGGATGGCCGACCGCCCGCCTGTG | GCCCGCCTGTGCCCATCA |
| rs34489327 | TYMS | ACGTTGGATGTGATGTAGAGTGTGGTTATG | ACGTTGGATGGGACGAATGCAGAACACTTC | TATTATAGCAACATATAAAACAACTAT |
| rs36210421 | KCNH2 | ACGTTGGATGACGTGGAGAGCAGGCTGGAT | ACGTTGGATGCACCTGCACTCCCTCACCT | CACCTGTTGAGCTGG |
| rs28399454 | CYP2A6 | ACGTTGGATGGTGATCCACGAGATCCAAAG | ACGTTGGATGGTGTCCTTTTTGACTCTGCG | ggatGGCCAAACTCATGGGGATCA |
| rs6791924 | SCN5A | ACGTTGGATGTCCCTGGCAGCCATCGAGAA | ACGTTGGATGCTCTCCTGCAAGGTGGTTG | atagCCTGCAAGGTGGTTGAGCCGC |
| rs3918290 | DPYD | ACGTTGGATGTCACTGAACTAAAGGCTGAC | ACGTTGGATGCACCAACTTATGCCAATTCTC | TTGTTTTAGATGTTAAATCACACTTA |
| rs7626962 | SCN5A | ACGTTGGATGAGTCGGCCTGAGATGCACTG | ACGTTGGATGGGATTCCAGGACCTGGAGC | TGTCAGCGACTGCCT |
| rs1801272 | CYP2A6 | ACGTTGGATGTCCCCTGCTCACCGCCAGT | ACGTTGGATGCATCGAGGAGCGCATCCAG | ccctGCTTCCTCATCGACGCCC |
| rs12721634 | CYP3A4 | ACGTTGGATGACTCACAGATAGAGGAGCAC | ACGTTGGATGTGATGGCTCTCATCCCAGAC | ccacGGAAACCTGGCTTCTCC |
| rs4986910 | CYP3A4 | ACGTTGGATGTGAAGGACTCTGATTAGAGC | ACGTTGGATGTTGGAAGTGGACCCAGAAAC | gAGAAACTGCATTGGCA |
| rs17238540 | HMGCR | ACGTTGGATGATGTATCACTCACCTCTAT | ACGTTGGATGTTGGACACAATGGATTAGGC | gtagTGGATTAGGCTGATATGAC |
