# Supplementary material for: Genetic polymorphisms of VIP variants in the Tajik ethnic group of northwest China
Source: BMC Genet. 2014 Sep 30;15:102. doi: 10.1186/s12863-014-0102-y (PMC4189671; doi:10.1186/s12863-014-0102-y)
Supplement: Additional file 2: — Genotype frequencies in Tajiks compared with four other populations. [file 12863_2014_102_MOESM2_ESM.docx]

| Additional file 2 Genotype frequencies in Tajiks compared with four other populations | | | | | | | | | | | | |
| --- | --- | --- | --- | --- | --- | --- | --- | --- | --- | --- | --- | --- |
| SNP ID | Gene | Category | | Allele | | Tajik Genotype Frequencies | | | *p* values against four populations (after Bonferroni correction) | | | |
|  |  | Family | Phase | A | B | AA(%) | AB(%) | BB(%) | CHB | JPT | CEU | YRI |
| rs1045642 | ABCB1 | ABC transporters | others | T | C | 34.3 | 45.5 | 20.2 | 8.49E-03 | 3.42E-02 | 3.10E-01 | 2.17E-18 |
| rs1128503 | ABCB1 | ABC transporters | others | T | C | 32.3 | 51.5 | 16.2 | 3.09E-02 | 8.73E-01 | 1.69E-02 | 3.03E-19 |
| rs2032582 | ABCB1 | ABC transporters | others | G | T | 18.0 | 49.4 | 32.6 | 9.02E-01 | 6.07E-01 | 8.24E-02 | - |
| rs2032582 | ABCB1 | ABC transporters | others | G | A | 72.7 | 27.3 | 0 | - | - | - | - |
| rs2032582 | ABCB1 | ABC transporters | others | T | A | 85.3 | 14.7 | 0 | - | - | - | - |
| rs975833 | ADH1A | alcohol dehydrogenase | phase I | G | C | 55.6 | 37.4 | 7.1 | 4.86E-16 | 3.45E-14 | 6.46E-01 | 5.79E-01 |
| rs1229984 | ADH1B | alcohol dehydrogenase | phase I | G | A | 48.0 | 45.0 | 7.0 | 3.76E-12 | 7.23E-11 | 9.18E-11 | 1.26E-10 |
| rs2066702 | ADH1B | alcohol dehydrogenase | phase I | C | T | 95.0 | 5.1 | 0 | 2.97E-01 | 2.97E-01 | 1.94E-01 | 1.61E-11 |
| rs698 | ADH1C | alcohol dehydrogenase | phase I | A | G | 44.3 | 45.4 | 10.3 | 1.21E-09 | 3.22E-09 | 1.24E-02 | 7.40E-11 |
| rs1801252 | ADRB1 | adrenergic receptors | others | G | A | 3.0 | 34.3 | 62.6 | 1.22E-05 | 1.52E-05 | - | 2.53E-06 |
| rs1801253 | ADRB1 | adrenergic receptors | others | C | G | 63.6 | 32.3 | 4.0 | 6.12E-01 | 3.88E-01 | 5.87E-02 | 4.12E-04 |
| rs1042713 | ADRB2 | adrenergic receptors | others | G | A | 36.0 | 49.0 | 15.0 | 4.49E-03 | 5.67E-01 | 7.34E-01 | 6.05E-02 |
| rs1042714 | ADRB2 | adrenergic receptors | others | G | C | 14.0 | 40.0 | 46.0 | 1.26E-03 | 3.39E-05 | 2.70E-02 | 9.85E-03 |
| rs1800888 | ADRB2 | adrenergic receptors | others | C | T | 96.0 | 4.0 | 0 | 4.84E-01 | 4.60E-01 | 5.86E-01 | 2.96E-01 |
| rs2066853 | AHR | AHR | others | G | A | 69.0 | 27.0 | 4.0 | 1.18E-05 | 7.65E-08 | 6.87E-02 | 9.06E-08 |
| rs4680 | COMT | COMT | phase II | A | G | 31.0 | 45.0 | 24.0 | 9.95E-05 | 1.56E-05 | 5.25E-01 | 3.15E-05 |
| rs1801272 | CYP2A6 | cytochrome P450 | phase I | T | / | 100 | 0 | 0 | - | 4.37E-01 | 1.40E-02 | - |
| rs28399433 | CYP2A6 | cytochrome P450 | phase I | G | T | 2.0 | 17.0 | 81.0 | - | - | - | - |
| rs28399444 | CYP2A6 | cytochrome P450 | phase I | A | / | 100 | 0 | 0 | - | - | - | - |
| rs28399454 | CYP2A6 | cytochrome P450 | phase I | G | / | 100 | 0 | 0 | - | - | 1.00E+00 | 7.81E-07 |
| rs3211371 | CYP2B6 | cytochrome P450 | phase I | C | T | 0 | 100 | 0 | - | - | - | - |
| rs3745274 | CYP2B6 | cytochrome P450 | phase I | G | T | 46.0 | 36.0 | 18.0 | 1.07E-03 | 2.03E-03 | 5.17E-02 | 1.50E-01 |
| rs28399499 | CYP2B6 | cytochrome P450 | phase I | T | / | 100 | 0 | 0 | - | - | - | 2.04E-06 |
| rs4244285 | CYP2C19 | cytochrome P450 | phase I | G | A | 85.0 | 15.0 | 0 | 1.00E-03 | 1.56E-05 | 4.07E-02 | 8.50E-02 |
| rs4986893 | CYP2C19 | cytochrome P450 | phase I | G | / | 100.0 | 0 | 0 | - | - | - | - |
| rs1799853 | CYP2C9 | cytochrome P450 | phase I | C | T | 100.0 | 0 | 0 | - | - | 6.25E-05 | - |
| rs16947 | CYP2D6 | cytochrome P450 | phase I | G | A | 49.4 | 49.4 | 1.2 | - | - | - | - |
| rs5030656 | CYP2D6 | cytochrome P450 | phase I | AAG | delAAG | 99.0 | 1.0 | 0 | - | - | - | - |
| rs28371706 | CYP2D6 | cytochrome P450 | phase I | C | T | 98.0 | 2.0 | 0 | - | - | - | - |
| rs28371725 | CYP2D6 | cytochrome P450 | phase I | G | A | 83.0 | 14.0 | 3.0 | - | - | - | - |
| rs59421388 | CYP2D6 | cytochrome P450 | phase I | C | / | 100 | 0 | 0 | - | - | - | - |
| rs61736512 | CYP2D6 | cytochrome P450 | phase I | C | / | 100 | 0 | 0 | - | - | - | - |
| rs890293 | CYP2J2 | cytochrome P450 | phase I | G | T | 0 | 97.0 | 3.0 | - | - | - | - |
| rs2740574 | CYP3A4 | cytochrome P450 | phase I | A | G | 97.0 | 3 | 0 | - | - | - | - |
| rs4986909 | CYP3A4 | cytochrome P450 | phase I | C | / | 100 | 0 | 0 | 1.00E+00 | 1.00E+00 | 1.00E+00 | - |
| rs4986910 | CYP3A4 | cytochrome P450 | phase I | T | / | 100 | 0 | 0 | - | - | 2.90E-01 | - |
| rs4986913 | CYP3A4 | cytochrome P450 | phase I | C | T | 98.0 | 2.0 | 0 | 1.00E+00 | 1.00E+00 | 7.13E-01 | 7.13E-01 |
| rs12721634 | CYP3A4 | cytochrome P450 | phase I | T | / | 100.0 | 0 | 0 | - | - | - | - |
| rs776746 | CYP3A5 | cytochrome P450 | phase I | G | A | 81.0 | 17.0 | 2.0 | 2.78E-05 | 8.74E-05 | 2.58E-02 | 1.23E-34 |
| rs10264272 | CYP3A5 | cytochrome P450 | phase I | C | / | 100 | 0 | 0 | 1.00E+00 | 1.00E+00 | - | 8.95E-09 |
| rs3918290 | DPYD | DPYD | phase I | G | / | 100 | 0 | 0 | - | - | 1.00E+00 | - |
| rs6277 | DRD2 | G-protein-coupled receptor | others | G | A | 38.0 | 47.0 | 15.0 | 7.41E-08 | 1.01E-07 | 1.98E-02 | 6.51E-11 |
| rs1800497 | DRD2 | G-protein-coupled receptor | others | T | C | 3.1 | 29.6 | 67.4 | 8.59E-06 | 1.43E-05 | 7.19E-01 | 2.25E-06 |
| rs6025 | F5 | F5 | others | C | A | 100 | 0 | 0 | - | - | 1.65E-01 | - |
| rs1695 | GSTP1 | glutathione S-transferase | phase II | A | G | 59.0 | 36.0 | 5.0 | 5.70E-01 | 1.96E-03 | 2.32E-04 | 1.50E-03 |
| rs1138272 | GSTP1 | glutathione S-transferase | phase II | T | C | 0 | 18.0 | 82.0 | 3.00E-03 | 2.00E-03 | 9.22E-01 | 2.28E-03 |
| rs3846662 | HMGCR | HMGCR | phase I | T | C | 18.0 | 61.0 | 21.0 | 2.49E-01 | 7.26E-01 | 2.12E-02 | 2.79E-24 |
| rs17238540 | HMGCR | HMGCR | phase I | T | / | 100 | 0 | 0 | - | - | - | - |
| rs17244841 | HMGCR | HMGCR | phase I | A | / | 100 | 0 | 0 | - | - | - | - |
| rs3807375 | KCNH2 | eag | others | A | G | 19.0 | 48.0 | 33.0 | 6.73E-07 | 8.34E-12 | 4.02E-01 | 1.13E-11 |
| rs3815459 | KCNH2 | eag | others | A | G | 16.0 | 49.0 | 35.0 | 6.04E-06 | 2.69E-09 | - | 6.88E-01 |
| rs12720441 | KCNH2 | eag | others | C | / | 100 | 0 | 0 | - | - | - | - |
| rs36210421 | KCNH2 | eag | others | G | T | 98.0 | 2.0 | 0 | - | - | - | - |
| rs5219 | KCNJ11 | inward-rectifier potassium channel | others | C | T | 36.7 | 38.8 | 24.5 | - | - | - | - |
| rs1801131 | MTHFR | methylenetetrahydrofolate reductase | phase I | C | A | 7.0 | 56.0 | 37.0 | 9.07E-03 | 5.46E-04 | 2.32E-01 | 7.36E-09 |
| rs1801133 | MTHFR | methylenetetrahydrofolate reductase | phase I | T | C | 4.0 | 30.3 | 65.7 | 3.13E-07 | 1.21E-03 | 1.92E-02 | 9.34E-03 |
| rs3814055 | NR1I2 | nuclear receptor | others | C | T | 30.0 | 56.0 | 14.0 | 8.60E-03 | 2.15E-03 | 1.00E-01 | 1.32E-03 |
| rs701265 | P2RY1 | G-protein coupled receptor | others | G | A | 4.0 | 32.0 | 64.0 | 7.61E-02 | 3.04E-01 | 9.33E-01 | 2.96E-25 |
| rs1065776 | P2RY1 | G-protein coupled receptor | others | T | C | 0 | 12.1 | 87.9 | - | - | - | - |
| rs2046934 | P2RY12 | G-protein coupled receptor | others | T | C | 82.0 | 16.0 | 2.0 | 5.69E-02 | 4.97E-02 | 4.49E-03 | 6.23E-03 |
| rs20417 | PTGS2 | nuclear receptor | others | G | C | 97.0 | 0 | 3.0 | 5.43E-04 | 1.57E-03 | 3.65E-07 | 4.82E-17 |
| rs689466 | PTGS2 | nuclear receptor | others | A | G | 73.7 | 23.2 | 3.0 | 3.68E-11 | 4.34E-07 | 7.13E-01 | 1.12E-01 |
| rs1805124 | SCN5A | sodium channel gene | others | G | A | 9.0 | 40.0 | 51.0 | 1.23E-04 | 7.59E-04 | 7.80E-03 | 4.96E-01 |
| rs6791924 | SCN5A | sodium channel gene | others | G | / | 100 | 0 | 0 | - | - | - | 2.00E-03 |
| rs7626962 | SCN5A | sodium channel gene | others | G | / | 100 | 0 | 0 | - | - | - | 1.24E-03 |
| rs12659 | SLC19A1 | solute carrier | others | C | T | 27.3 | 58.6 | 14.1 | - | - | - | - |
| rs1051266 | SLC19A1 | solute carrier | others | G | A | 29.9 | 51.6 | 18.6 | 3.65E-01 | 8.28E-02 | 8.36E-01 | 2.83E-06 |
| rs1131596 | SLC19A1 | solute carrier | others | T | C | 37.5 | 46.3 | 16.3 | - | - | - | - |
| rs4149056 | SLCO1B1 | solute carrier | others | T | C | 82.0 | 17.0 | 1.0 | 2.30E-01 | 7.68E-01 | 1.47E-01 | 2.66E-04 |
| rs3760091 | SULT1A1 | sulfotransferase | phase II | C | G | 27.6 | 53.1 | 19.4 | - | - | - | - |
| rs1801030 | SULT1A2 | sulfotransferase | phase II | A | / | 100 | 0 | 0 | - | - | - | - |
| rs1142345 | TPMT | methyltransferase | phase II | G | A | 0 | 0 | 100 | 1.00E+00 | 6.94E-01 | 5.50E-02 | 1.56E-02 |
| rs1800460 | TPMT | methyltransferase | phase II | A | G | 0 | 0 | 100 | - | - | - | - |
| rs34489327 | TYMS | TYMS | phase II | DEL | / | 100 | 0 | 0 | - | - | - | - |
| rs4124874 | UGT1A10 | UDP-glucuronosyltransferase | phase II | C | A | 14.4 | 56.7 | 28.9 | 1.64E-02 | 6.88E-02 | 3.31E-01 | 2.73E-23 |
| rs4148323 | UGT1A10 | UDP-glucuronosyltransferase | phase II | A | G | 0 | 7.0 | 93.0 | 7.23E-08 | 3.22E-03 | 9.00E-02 | 9.00E-02 |
| rs10929302 | UGT1A10 | UDP-glucuronosyltransferase | phase II | G | A | 53.0 | 41.0 | 6.0 | 2.58E-03 | 3.31E-03 | 9.38E-01 | 4.91E-02 |
| rs1540339 | VDR | nuclear receptor | others | G | A | 47.5 | 39.4 | 13.1 | 1.60E-10 | 1.42E-11 | 7.78E-01 | 1.80E-02 |
| rs1544410 | VDR | nuclear receptor | others | G | A | 40.0 | 52.0 | 8.0 | 3.05E-12 | 2.03E-06 | 1.75E-02 | 2.50E-01 |
| rs2228570 | VDR | nuclear receptor | others | T | C | 15.0 | 39.0 | 46.0 | - | - | - | - |
| rs2239179 | VDR | nuclear receptor | others | A | G | 31.0 | 51.0 | 18.0 | 4.01E-04 | 6.14E-05 | 3.63E-02 | 8.58E-03 |
| rs2239185 | VDR | nuclear receptor | others | T | C | 22.0 | 58.0 | 20.0 | 2.86E-04 | 1.55E-01 | - | 4.37E-01 |
| rs3782905 | VDR | nuclear receptor | others | C | G | 46.0 | 48.0 | 6.0 | 1.20E-01 | 6.72E-04 | 3.92E-01 | 7.74E-02 |
| rs7975232 | VDR | nuclear receptor | others | C | A | 20.0 | 58.0 | 22.0 | 9.30E-05 | 2.13E-03 | 1.92E-02 | 2.44E-02 |
| rs10735810 | VDR | nuclear receptor | others | C | T | 48.4 | 36.3 | 15.4 | 8.17E-02 | 8.74E-01 | 2.72E-01 | 4.64E-03 |
| rs11568820 | VDR | nuclear receptor | others | G | A | 60.6 | 33.3 | 6.1 | 1.12E-04 | 4.25E-05 | 6.86E-01 | 8.35E-38 |
| rs7294 | VKORC1 | VKORC1 | phase I | C | T | 44.0 | 46.0 | 10.0 | 8.00E-10 | 1.09E-06 | 7.69E-01 | 1.59E-04 |
| rs9934438 | VKORC1 | VKORC1 | phase I | G | A | 26.0 | 49.0 | 25.0 | 1.29E-17 | 4.64E-14 | 1.01E-01 | 9.39E-25 |
